# miR-134-3p Regulates Cell Proliferation and Apoptosis by Targeting INHBA via Inhibiting the TGF-β/PI3K/AKT Pathway in Sheep Granulosa Cells

**DOI:** 10.3390/biology14010024

**Published:** 2024-12-30

**Authors:** Xinai Huang, Yongjin Bao, Fan Yang, Xiaodan Li, Feng Wang, Chunxiang Zhang

**Affiliations:** 1 College of Animal Science, Shanxi Agricultural University, Taigu 030801, China; 2 Jiangsu Livestock Embryo Engineering Laboratory, Nanjing Agricultural University, Nanjing 210095, China; baoyongjin666888@163.com (Y.B.); 18061284040@163.com (F.Y.); lixd@jsafc.edu.cn (X.L.); caeet@njau.edu.cn (F.W.)

**Keywords:** miR-134-3p, INHBA, proliferation, apoptosis, sheep granulosa cells

## Abstract

Inhibin β-A (*INHBA*), a TGF-β superfamily member, is crucial for developing follicles. Although miRNAs are essential for post-transcriptional gene regulation, it is not yet known how they affect the expression of *INHBA* during follicle development. Using bioinformatics analyses, miR-134-3p was found, in this investigation, to be a crucial microRNA that targets *INHBA* in sheep GCs. Furthermore, when the follicular diameter expanded, there was a discernible decline in miR-134-3p expression. The miR-134-3p overexpression markedly reduced the proliferation of GCs, whereas its knockdown augmented it. Moreover, cell cycle progression was enhanced by miR-134-3p overexpression. Furthermore, miR-134-3p overexpression heightened GC apoptosis, while its knockdown reduced it. Importantly, miR-134-3p overexpression blocked the PI3K/AKT/mTOR axis, whereas its knockdown stimulated it. Overall, the outcomes of transfections with *INHBA* and miR-134-3p showed that, in sheep GCs, miR-134-3p targets *INHBA* to control cell proliferation and apoptosis. In summary, these results add to our understanding of the molecular mechanisms involving important miRNAs in ewe fecundity by indicating that miR-134-3p influences cell proliferation, cell apoptosis, and the TGF-β/PI3K/AKT/mTOR axis, which, in turn, influences the follicular development of sheep GCs.

## Simple Summary

Normal folliculogenesis is essential for mammalian fertility. Ovarian granulosa cells (GCs) are necessary in mammalian reproduction as they secrete factors promoting oocyte growth and maturation. Although the role of inhibitin beta A (*INHBA*) in the proliferation and secretion functions of GCs is well established, the post-transcriptional regulation of *INHBA* expression during folliculogenesis in sheep remains unclear. This research investigated the role of miRNAs in sheep GCs that target *INHBA* and assessed the potential functions of miR-134-3p in the activity of GCs in sheep. These results confirmed the functions of miR-134-3p in controlling GC proliferation and apoptosis via influencing the TGF-β/PI3K/AKT pathway.

## 1. Introduction

A key economic goal in the sheep industry is to improve reproductive efficiency, particularly litter size (fecundity). However, most sheep breeds typically give birth to a single lamb, with only a small percentage delivering twins. The intensive management of Hu sheep has led to the selection of larger litters and the fixation of the FecB mutation in BMPR1B, resulting in high prolificacy [1]. Hu sheep are thus recognized as good models for investigating reproductive traits in prolific breeds. The interest in genes and non-coding RNAs (ncRNAs) correlated with fertility has increased due to the poor heritability of litter size, which challenges conventional selection methods. GCs are essential for follicle development, as they support the developing oocyte, proliferate, and secrete sex steroids and growth factors. The exploration of GC-associated pathways and functions, therefore, enhances the understanding of follicular development [2].

The TGF-β superfamily members, activin and inhibin, secreted by GCs, respectively, stimulate and inhibit the pituitary’s production of follicle-stimulating hormones (FSHs). These proteins act as crucial autocrine and paracrine regulators of follicular function [3]. Depending on the differentiation stage of granulosa cells, activins and inhibins can either enhance or suppress progesterone and estradiol production [4]. The functions of activins in ovulation, steroidogenesis, atresia, follicular expansion, and primordial germ cell development are well-documented [5]. Inhibins counteract activins by competitive interactions with type II activin receptors, blocking activin signaling [6,7]. Activins (A, B, and AB) are formed by the dimerization of inhibin β subunits (A/B/C/D and/or E). Inhibins represent heterodimers of α- and β-subunits, forming either inhibin A or inhibin B [8]. Activin A consists of two disulfide-linked *INHBA* subunits, whereas inhibin A comprises an INHBA subunit and an INHA subunit. Porcine GCs with an increased *INHBA* expression produce more activin, lowering GC apoptosis and follicular atresia [9]. Decreased levels of *INHBA* in Hu sheep have been associated with reduced fecundity, resulting in inhibited GC proliferation and increased apoptosis [10,11]. The post-transcriptional regulation of *INHBA* expression in sheep GCs, however, remains poorly understood.

MicroRNA (miRNA) is the most extensively studied type of ncRNA involved in modulating sheep follicle development [12,13,14]. The elucidation of miRNA networks can clarify the molecular pathways involved in follicle development, as well as offer new opportunities for treating ovarian dysfunctions [15]. Apoptosis and proliferation are two important granulosa cell biological processes modulated by certain miRNAs that interact with the TGF-β axis [2]. A bioinformatic investigation has found 3 miRNAs (miR-136, miR-374a, and miR-9-5p) that co-regulate the *INHBA* and *EREG* genes, which contribute to high levels of reproduction in Finn sheep [12]. A new study has identified many miRNAs involved in the growth of ewe follicles stimulated by FSH, including miR-181a-5p-INHBA and miR-27a-3p-INHBA, which may affect processes such as apoptosis, proliferation, steroidogenesis, and differentiation [16]. Therefore, exploring the involvement of miRNAs in sheep GCs that target *INHBA* will help identify the key regulators of sheep fertility.

Here, bioinformatics analyses, was utilized to discover that miR-134-3p may control *INHBA* by targeting the 3′-UTR of *INHBA* in sheep granulosa cells. Following that, it was found that miR-134-3p blocks the PI3K/AKT/mTOR axis, lowers proliferation, and causes apoptosis in sheep GCs. Furthermore, according to the present results, via blocking the PI3K/AKT axis in sheep GCs, miR-134-3p modulates proliferation and apoptosis in these cells.

## 2. Materials and Methods

### 2.1. Ethical Approval and Reagents

Each animal-related study was carried out following the rules set forth by Nanjing Agricultural University’s Ethics Committee in China (Approval No. SYXK2022-0031). Unless otherwise specified, all chemicals were manufactured by Life Technologies (Carlsbad, CA, USA).

### 2.2. Bioinformatics Predictions of miRNAs That Target INHBA

First, the sheep *INHBA* gene’s 3′-UTR sequence was acquired from the ARS-UI_Ramb_v2.0 assembly’s Entrez Nucleotide database of chromosome 4. The online databases miRanda, RNAhybrid, and miRBase were then utilized to predict putative miRNAs that target *INHBA*.

### 2.3. Plasmids and Luciferase Reporter Assays

To verify the intended association between the proposed miRNAs and *INHBA* mRNA, dual-luciferase reporter experiments were performed. In the plasmid GP-miRGLO vectors (Tsingke, Beijing, China), the *INHBA* 3′-UTR sequences, including the wild or mutant putative miRNA target sites, were cloned. Tsingke Biological Technology sequenced all recombinant vectors and mimicked miRNAs to ensure proper insertion. Interactions were measured using a Dual-Luciferase^®^ Reporter Assay System (DL101, Vazyme, Nanjing, China), according to the directions provided, after co-transfection in 293T cells for 48 h. Table 1 and Table 2 list the miRNA and *INHBA*-3′-UTR vector sequences. The *INHBA* overexpression vector used the previously constructed vector [10].

### 2.4. Collection of Samples

Ovaries were harvested from sexually mature Hu ewes at a neighboring abattoir in Taicang, Jiangsu (31°45′ N, 121°10′ E) during the December–January breeding season. The tissues and GCs were collected as described, with slight modifications [17]. A caliper was used to separate the visible antral follicles into 3 size groups (diameters of ≤3, 3–5, and ≥5 mm) after the ovaries had been cleaned. While some follicles were used to separate granulosa cells, others were instantly frozen in liquid nitrogen.

### 2.5. Culture of GCs

For in vitro culture investigations, granulosa cells were specifically acquired from follicles ≥ 3 mm. A previously described strategy was employed to isolate GCs. It should be mentioned that follicular GCs spontaneously transform into luteinized GCs following a 48 h culture with serum. First-passage GCs were employed in later tests, with further GC propagation. Isolated GCs (5 × 10^5/^well) were inoculated in 6-well plates that contained DMEM/F12 along with 10% FBS. After selecting cells in a healthy logarithmic growth phase, they were rinsed with PBS and harvested with trypsin, and the supernatant was removed by centrifugation. A freezing medium was combined with 70% complete culture medium, 20% fetal bovine serum, and 10% DMSO. After the adjustment of the density to (1–10) × 10^6^/mL, cells were placed in a freezing medium. The cell suspension was aliquoted into cryovials, with 1 mL aliquoted per vial. The cryovials were labelled with the type of cells and date of freezing, and thencooled in the following order: room temperature → 4 °C (30 min) → −20 °C (60 min) → −80 °C overnight → liquid nitrogen. After thawing at 37 °C, cells were placed in a centrifuge tube with more than ten times the volume of the culture medium. The tube was mixed thoroughly, then centrifuged and the supernatant removed; the culture medium was added to the pellet and the culturing process proceeded. Following the provided protocols, the cultured GCs were transfected with plasmid vectors, siRNAs, and/or miRNA mimics using lipofectamine 3000 once they reached 70–80% confluence for each treatment. The cells were taken out for further examination after growing for 24 or 48 h at 37 °C with 5% CO_2_.

### 2.6. Gene Expression Analysis Using qRT-PCR

Total RNA was extracted withTRIzol as instructed. Electrophoresis and an ND-2000 spectrophotometer (Thermo Fisher, Waltham, MA, USA) were used to assess the extracted RNA samples. The RNA samples were reverse-transcribed to cDNA employing HiScript II Q Select RT SuperMix with gDNA Wiper (R233-01; Vazyme, Nanjing, China). The miRNA First Strand cDNA Synthesis Kit (by stem-loop) (MR101-01; Vazyme) was used for miRNA reverse transcription.

qRT-PCR was conducted with ChamQTM Universal SYBR^®^ qPCR Master mix (Q711-02; Vazyme) on an ABI 7500 Real-Time PCR System (Applied Biosystems, Foster City, CA, USA). The primer specificities for genes were verified using Sanger sequencing, the electrophoresis of PCR products, and BLAST alignments. The details of each primer sequence used are given in Table 3 and Table 4. The R^2^ values for all qRT-PCR standard curves were 0.992 to 0.999, with the PCR efficiency ranging from 95% to 105%. Each PCR run incorporated 3 negative controls, specifically no template controls. The relative miRNA levels were determined as 2^−∆∆CT^ values, using GAPDH for normalization.

### 2.7. Protein Expression Analysis Using Western Blot Assays

The relative protein expression was assessed following our prior methodology, with slight adjustments [18], employing the GAPDH protein for normalization. The relevant primary antibodies included -AKT, mouse anti-GAPDH, -P-mTOR, -mTOR, -P-AKT, -TGFβR2, -TGFβ1, -SMAD2, -PCNA, -BAX, and -BCL2, and rabbit anti-INHBA. The secondary antibodies were goat anti-mouse and anti-rabbit IgG. GAPDH represented the loading control. Table 5 contains the specific information on all of the antibodies used. Importantly, all primary antibodies were tested for sheep proteins before being used in our experiments.

### 2.8. EdU and CCK-8 Assays

Proliferation was evaluated using CCK-8 kits (KGA317, KeyGen, Nanjing, China). In short, each replica received 10 µL of CCK-8 reagent for 4 h at 37 °C before the reading of absorbances at 450 nm. The kFluor555 Click-iT EdU kit (KGA337-100, KeyGen, Nanjing, China) was also used to measure cell proliferation relative to EdU-positive cells, following the manufacturer’s instructions. EdU incorporation was observed through a fluorescent microscope (Olympus, Tokyo, Japan).

### 2.9. Cell Cycle and Apoptosis Evaluations

These evaluations were conducted as described [19]. Apoptosis was assessed using a FITC Annexin V Apoptosis Detection Kit I (C1062M, Beyotime, Shanghai, China). A cell cycle detection kit (C6031S, Bioscience, Shanghai, China) was utilized to describe the cell cycle distribution, following the directions provided by the manufacturer. The FlowJo (10.7.1 for Windows) software was used to save and process all flow cytometry (BD Biosciences, San Jose, CA, USA) data.

### 2.10. Statistical Analysis

With a minimum of three biological replicates, each experiment was conducted three times. The SPSS statistical software (v.26.0 for Windows) was used for statistical analyses. The t-test was utilized to compare two independent groups. Tukey’s post hoc test was applied after a one-way ANOVA comparing more than two groups. The mean ± standard error of the mean (S.E.M.) is used for data presentation. A significant probability value was defined as *p* < 0.05.

## 3. Results

### 3.1. miR-134-3p Targets INHBA Regulation

**Figure 1:**
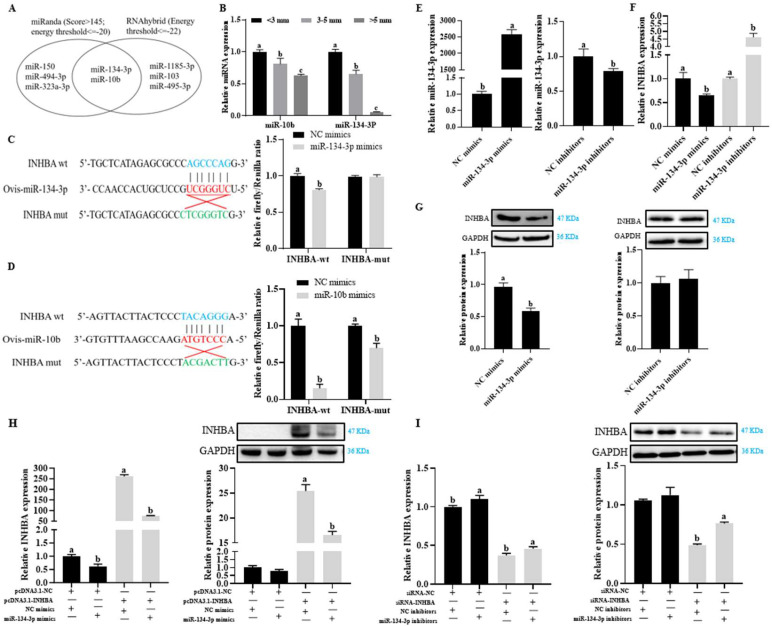
miR-134-3p targets *INHBA* regulation. (**A**) miR-134 indicates that *p* and miR-10b are anticipated to target *INHBA,* according to RNAhybrid and miRanda. (**B**) GC levels of predicted miRNAs (miR-134-3p and miR-10b) in follicles <3, 3–5, and >5 mm. qPCR analysis was used to identify the miRNA expression data, which were then normalized to miR-16b. (**C**,**D**) Diagrams showing how miR-134-3p and miR-10b interact with both mutant (mut) and wild-type (wt) *INHBA* (blue and green, respectively). Red nucleotides indicate the miR-134-3p and miR-10b seed sequences. Co-transfection of reporter vectors for wt or mut miR-134-3p-*INHBA* interaction site and miR-10b or NC mimics in 293T cells (right) and luciferase assays. (**E**) Levels of miR-134-3p with transfection of NC mimics, miR-134-3p mimics, NC inhibitors, or miR-134-3p inhibitors. (**F**,**G**) Relative *INHBA* expression following transfection with NC mimics, miR-134-3p mimics, NC inhibitors, or miR-134-3p inhibitors, respectively. (**H**) *INHBA* mRNA and protein levels with vector-null and NC mimics, vector-null and miR-134-3p mimics, *INHBA* overexpression and NC mimics, *INHBA* overexpression and miR-134-3p mimics co-transfection. (**I**) *INHBA* mRNA and protein levels with siRNA-NC and NC mimics, siRNA-NC and miR-134-3p mimics, *INHBA* suppression and NC mimics, *INHBA* suppression and miR-134-3p inhibitors co-transfection. Data are given as mean ± SEM. Distinct letters (a, b, and c) denote significant differences (*p* < 0.05).

Putative upstream miRNAs of *INHBA* were identified through bioinformatics analyses using RNAhybrid and miRanda tools. Only two widely conserved miRNAs, miR-134-3p and miR-10b, were identified as common across the databases (Figure 1A). The expression levels of these two miRNAs showed a significant decrease with increasing follicle size (*p* < 0.05, Figure 1B). A dual-luciferase reporter vector was constructed to investigate whether *INHBA* is a direct target of these miRNAs, incorporating wild-type or mutated putative miRNA response elements (MREs) in the 3′-UTR of *INHBA*. Luciferase assays demonstrated that the mutation of miR-134-3p within the *INHBA*-MRE led to a significant loss of activity when compared to the wild-type *INHBA* reporter (*p* < 0.05, Figure 1C,D). Thus, miR-134-3p was selected for further studies due to its direct targeting of *INHBA* mRNA. The efficacy of miR-134-3p mimics and inhibitors was verified in Hu sheep GCs (*p* < 0.05, Figure 1E). Following the transfection of sheep GCs with miR-134-3p mimics or inhibitors, a significant inverse relationship was observed between the level of *INHBA* mRNA and that of miR-134-3p (*p* < 0.05, Figure 1F). Only miR-134-3p mimics markedly reduced the protein levels of INHBA (*p* < 0.05, Figure 1G and Figure S1A). The co-transfection with miR-134-3p mimics reduced the overexpression effect of pcDNA3.1-*INHBA* on *INHBA* levels (*p* < 0.05, Figure 1H and Figure S1B). When miR-134-3p inhibitors were co-transfected, the knockdown effect of siRNA-*INHBA* on *INHBA* levels was reduced (*p* < 0.05, Figure 1I and Figure S1C). The findings demonstrate that miR-134-3p interacts directly with the *INHBA* mRNA 3′-UTR, resulting in the downregulation of its expression in small and medium GCs.

### 3.2. miR-134-3p Inhibits Proliferation in Sheep GCs

**Figure 2:**
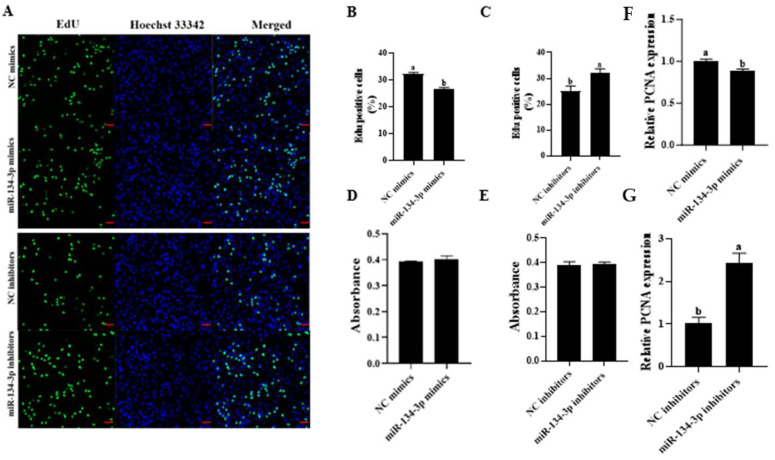
miR-134-3p blocks proliferation in sheep GCs. Analysis of cell proliferation was conducted through EdU (**A**–**C**) and CCK-8 (**D**,**E**) assays (scale bar = 50 µm). (**F**,**G**) PCNA mRNA levels. The mean ± standard error of the mean (S.E.M.) is used for data presentation. Data are given as mean ± SEM. Distinct letters (a, b) denote significant differences (*p* < 0.05).

**Figure 3:**
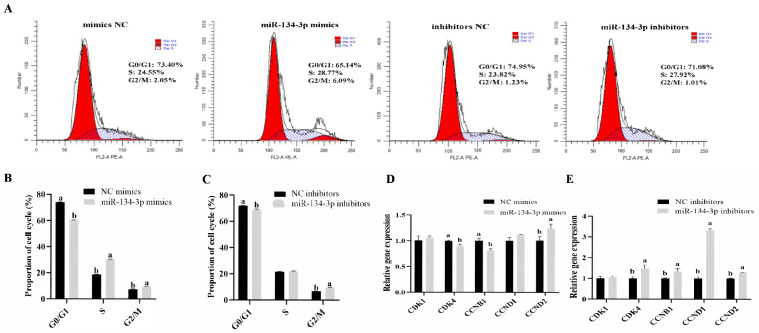
Influence of miR-134-3p on the GC cell cycle. (**A**–**C**) Impact of miR-134-3p on the cell cycle in GCs following transfection with miR-134-3p mimics or inhibitors. (**D**,**E**) Levels of cell cycle-associated genes following transfection. Data are given as mean ± SEM. Distinct letters (a, b) denote significant differences (*p* < 0.05).

The EdU assay demonstrated that miR-134-3p overexpression markedly reduced the proliferation of sheep GCs, while miR-134-3p silencing enhanced it (*p* < 0.05, Figure 2A–C). According to the CCK-8 assay, miR-134-3p had no discernible influence on the GCs’ growth rate (*p* < 0.05, Figure 2D,E). miR-134-3p markedly lowered PCNA mRNA, while the silencing of miR-134-3p raised it (*p* < 0.05, Figure 2F,G). Both miR-134-3p mimics and inhibitors raised cell proportions in G2/M but reduced those in G0/G1, as shown by flow cytometry. Only miR-134-3p mimics led to increases in cell proportions in the S phase (*p* < 0.05, Figure 3A–C). miR-134-3p consistently lowered *CDK4* and *CCNB1* mRNAs while elevating the mRNA levels of *CCND2* (*p* < 0.05, Figure 3D). In contrast, the application of miR-134-3p inhibitors led to marked reductions in *CDK4*, *CCNB1*, *CCND1*, and *CCND2* mRNA (*p* < 0.05, Figure 3E). Thus, miR-134-3p blocks proliferation in sheep GCs.

### 3.3. miR-134-3p Promotes Apoptosis in Sheep GCs

**Figure 4:**
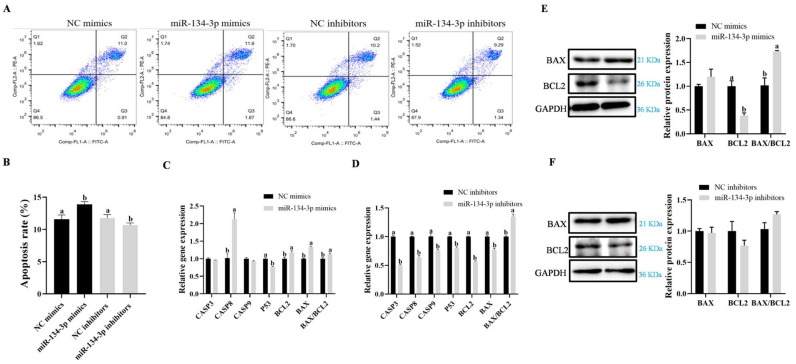
GC apoptosis is promoted by miR-134-3p. (**A**,**B**) The percentage of apoptosis (total apoptosis = late and early apoptosis) in GCs with overexpression and silencing of miR-134-3p is displayed by flow cytometry. (**C**,**D**) miR-134-3p overexpression and inhibition in the mRNA expression of genes associated with apoptosis. (**E**,**F**) Expression of the proteins BAX and BCL2. Data are given as mean ± SEM. Distinct letters (a, b) denote significant differences (*p* < 0.05).

According to flow cytometry analysis, GC apoptosis was markedly raised in cells expressing miR-134-3p mimics but was reduced after the exposure to inhibitors (*p* < 0.05, Figure 4A,B). While downregulating the mRNA levels of *P53*, the overexpression of miR-134-3p markedly elevated *CASP8*, *BAX*, and *BCL2* mRNAs and the *BAX*/*BCL2* ratio. On the other hand, the mRNA expression of *CASP3*, *CASP8*, *CASP9*, *P53*, *BAX*, and *BCL2* was significantly reduced when miR-134-3p was suppressed, although the *BAX*/*BCL2* ratio increased (*p* < 0.05, Figure 4C,D). In comparison, there were no discernible changes in theBAX, BCL2, or BAX/BCL2 proteins (*p* <0.05, Figure 4E,F and Figure S2A,B), while the overexpression of miR-134-3p led to a marked drop in BCL2 protein and an increase in the BAX/BCL2 ratio. These findings imply that, in sheep GCs, miR-134-3p stimulates apoptosis.

### 3.4. miR-134-3p Suppresses the TGF-β/PI3K/AKT Axis

**Figure 5:**
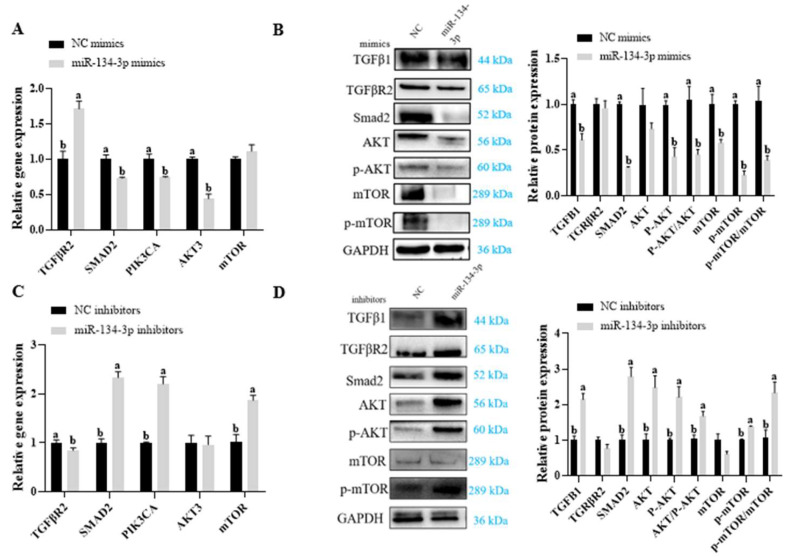
miR-134-3p suppresses the TGF-β/PI3K/AKT axis**.** mRNA levels of PI3K/AKT-related components after miR-134-3p mimic (**A**) and miR-134-3p inhibitor (**C**) exposure. Levels of PI3K/AKT-related proteins following miR-134-3p mimic (**B**) and miR-134-3p inhibitor (**D**) exposure. Data are given as mean ± SEM. Distinct letters (a, b) denote significant differences (*p* < 0.05).

Given the importance of *INHBA* in TGF-β activities and the critical function of the PI3K/AKT axis in cell growth, this work examined whether miR-134-3p influences GC proliferation and apoptosis by mediating TGF-β/PI3K/Akt signaling transduction. First, the impact of miR-134-3p levels on the TGF-β/PI3K/AKT axis in sheep GCs was evaluated. While *SMAD2*, *PIK3CA*, and *AKT3* mRNA levels decreased, miR-134-3p overexpression significantly elevated *TGFβR2* expression. Furthermore, it caused a decrease in the protein levels of several proteins associated with TGF-β/PI3K/AKT signaling, including *TGFβ1*, *SMAD2*, P-AKT, mTOR, and p-mTOR (*p* < 0.05, Figure 5A,B and Figure S3A). On the other hand, *SMAD2*, *PIK3CA*, and *mTOR* mRNA levels increased while TGFβR2 decreased markedly when miR-134-3p was interfered with. Furthermore, it enhanced the levels of the TGF-β1, SMAD2, AKT, p-AKT, and p-mTOR proteins (*p* < 0.05, Figure 5C,D and Figure S3B). These findings imply that, in sheep GCs, miR-134-3p suppresses the TGFβ/PI3K/AKT axis.

### 3.5. miR-134-3p Modulates Proliferation and Apoptosis by Targeting INHBA in Sheep GCs

**Figure 6:**
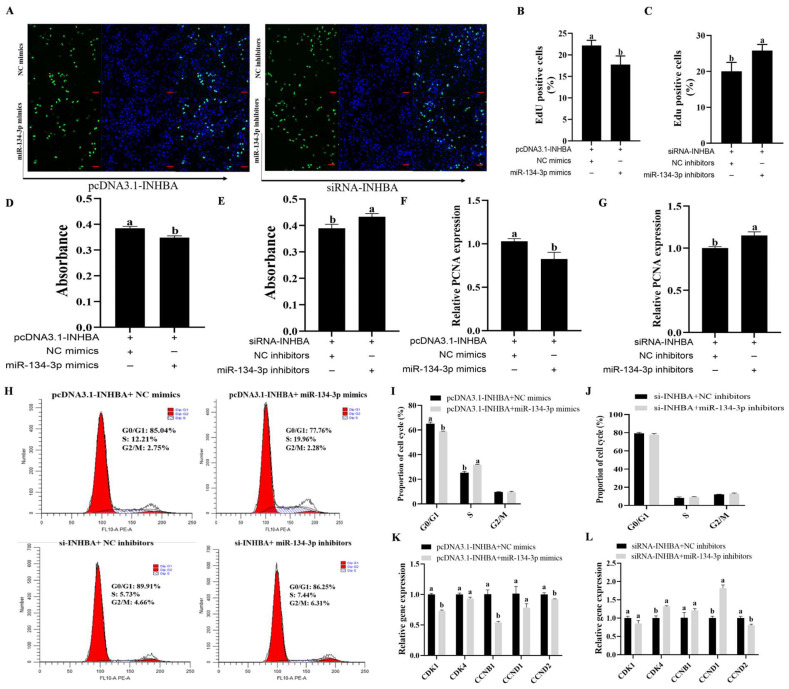
miR-134-3p regulates GC proliferation by targeting *INHBA*. *INHBA* suppression and miR-134-3p inhibition, as well as the combination of *INHBA* and miR-134-3p overexpression, all affect GC proliferation. Analysis of cell proliferation using CCK-8 (**D**,**E**) and EdU incorporation assays (**A**–**C**) (corresponding scale bars = 50 µm) (**F**,**G**) PCNA mRNA levels. (**H**–**J**) Effects of *INHBA* and miR-134-3p overexpression, *INHBA* suppression and miR-134-3p inhibition on the GC cell cycle. (**K**,**L**) Levels of cell cycle-related gene GCs overexpressing *INHBA* and miR-134-3p, *INHBA* suppression and miR-134-3p inhibition. Data are given as mean ± SEM. Distinct letters (a, b) denote significant differences (*p* < 0.05).

**Figure 7:**
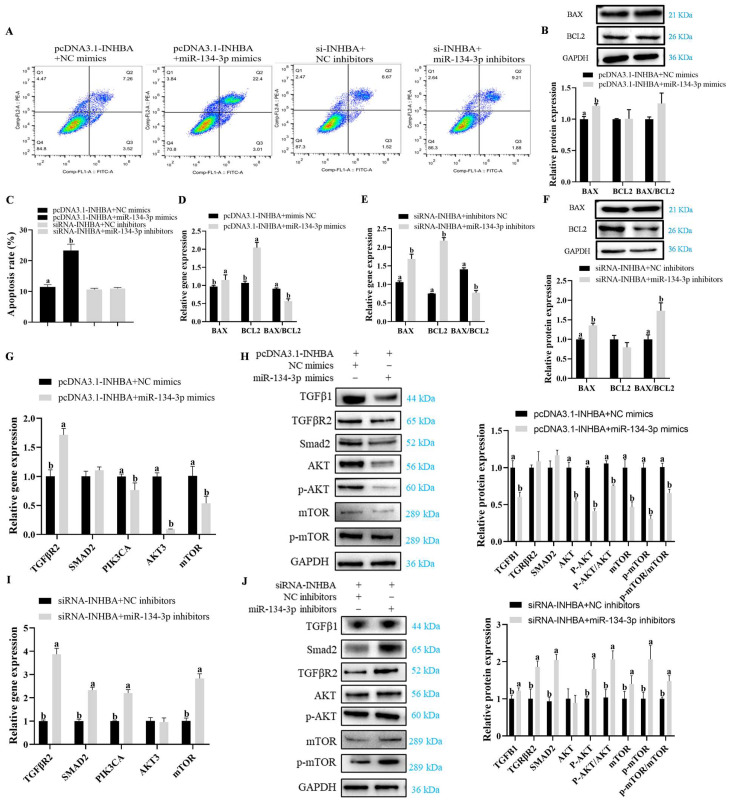
miR-134-3p modulates apoptosis in GCs and influences the TGF-β/PI3K/AKT axis via *INHBA*. Flow cytometry illustrating the effect of *INHBA* overexpression and miR-134-3p mimics co-transfection, *INHBA* silencing and miR-134-3p co-transfection on GC apoptosis. (**A**,**C**) The apoptosis percentage in GCs is displayed by flow cytometry (total apoptosis = late and early apoptosis). (**D**,**E**) *INHBA* overexpression and miR-134-3p mimics co-transfection, *INHBA* silencing and miR-134-3p co-transfection, and apoptosis-related mRNA levels (**B**,**F**) Levels of BAX and Bcl2 proteins. mRNA levels of PI3K/AKT axis-related genes with pcDNA3.1-*INHBA* and miR-134-3p mimics co-transfection (**G**), and siRNA-*INHBA* and miR-134-3p inhibitors co-transfection (**I**) treatment. Protein expression of PI3K/AKT pathway-related protein with pcDNA3.1-*INHBA* and miR-134-3p mimics co-transfection (**H**), and siRNA-*INHBA* and miR-134-3p inhibitors co-transfection (**J**) treatment. Data are given as mean ± SEM. Distinct letters (a, b) denote significant differences (*p* < 0.05).

Co-transfection experiments were carried out using pcDNA3.1-*INHBA* and miR-134-3p mimics or si*INHBA* and miR-134-3p inhibitors to assess their effects on GC proliferation and apoptosis through the targeting of *INHBA* by miR-135-3p. When pcDNA3.1-*INHBA* and miR-134-3p mimics were co-transfected, GC proliferation was reduced, while when si*INHBA* and miR-134-3p inhibitors were transfected, it was increased (Figure 6A–E). Similarly, the co-transfection with pcDNA3.1-*INHBA* and miR-134-3p mimics markedly reduced *PCNA* mRNA, whereas that with si*INHBA* and miR-134-3p inhibitors upregulated it (Figure 6F,G). Furthermore, the co-transfection of si*INHBA* and miR-134-3p inhibitors did not have marked effects on cell cycle proportions, though the combination of miR-134-3p mimics and pcDNA3.1-*INHBA* led to greater numbers of cells in the S-phase, with fewer in G0/G1 (*p* < 0.05, Figure 6I,J). *CDK1*, *CCNB1*, and *CCND2* mRNA expression was considerably decreased after the co-transfection with pcDNA3.1-*INHBA* and miR-134-3p mimics (*p* < 0.05, Figure 6K). On the contrary, the combination of si*INHBA* and miR-134-3p inhibitors markedly raised *CDK4* and *CCND1* mRNA levels while lowering *CCND2* mRNA expression (*p* < 0.05, Figure 6L). The co-transfection with pcDNA3.1-*INHBA* and miR-134-3p mimics considerably raised the proportion of apoptotic GCs, according to flow cytometry studies. However, there was no discernible change in this percentage when si*INHBA* and miR-134-3p inhibitors were co-transfected (*p* < 0.05, Figure 7A,C). Furthermore, the combination of si-*INHBA* and miR-134-3p inhibitors or pcDNA3.1-*INHBA* and miR-134-3p mimics markedly raised the levels of *BAX* and *BCL2* mRNA while decreasing the BAX/BCL2 ratio (*p* < 0.05, Figure 7D,E) and increasing BAX protein levels (*p* < 0.05, Figure 7B,F and Figure S4A,B). The combination of si*INHBA* and miR-134-3p inhibitors markedly enhanced the BAX to BCL2 protein ratio (*p* < 0.05, Figure 7F).

The effects of these co-transfections on the TGF-β/PI3K/AKT axis were investigated. qRT-PCR and Western blotting demonstrated that the co-transfection with pcDNA3.1-*INHBA* and miR-134-3p mimics markedly upregulated TGFβR2 and downregulated *PIK3CA*, *AKT3*, and *mTOR*. Furthermore, the TGFβ1, AKT, P-AKT, P-AKT/AKT, mTOR, P-mTOR, and P-mTOR/mTOR protein levels were reduced (*p* < 0.05, Figure 7G,H and Figure S5A). In contrast, the combination of *siINHBA* and miR-134-3p inhibitors markedly raised the mRNA levels of *TGFβR2*, *SMAD2*, *PIK3CA*, and *mTOR*, in addition to upregulating the protein levels of a large number of PI3K/AKT axis components (*p* < 0.05, Figure 7I,J and Figure S5B). The findings indicate that miR-134-3p modulates proliferation and apoptosis in GCs via *INHBA*, together with the modulation of the TGF-β/PI3K/Akt axis.

## 4. Discussion

According to earlier research, *INHBA* in ovarian GCs influence GC proliferation, apoptosis, and hormone synthesis [9,10,16,20], as well as follicle growth [9,10]. The post-transcriptional regulation of α-subunits (INHA) and β-subunits (INHBA and INHBB) which control the synthesis of inhibin and activin is not well understood [9]. MiRNA has been found to control follicular development through the canonical targeting and translational repression of specific genes [21]. The present research also examined the molecular processes of *INHBA* in Hu sheep GCs using bioinformatic analyses. It found that miR-134-3p is a potential modulator of *INHBA* that targets the *INHBA* 3′-UTR. It was found that miR-134-3p levels decreased with increasing follicle diameter, similar to our prior research indicating that *INHBA* expression rises with follicle diameter in sheep ovaries [10]. The results revealed that miR-134-3p blocks proliferation directly while promoting apoptosis in sheep GCs by suppressing *INHBA* expression and regulating follicular development. Furthermore, as some of the miRNAs that target *INHBA* have already been found in sheep follicles (miR-181a-5p, miR-182-5p, and miR-27a-3p) [16] and porcine GCs (miR-214-5p, miR-7144-3p, and miR-9830-5p) [9], these findings demonstrate that the influence of miR-134-3p mimics is more effective on INHBA protein expression than that on miR-134-3p inhibition in this pathway. This demonstrates that miR-134-3p regulates follicular development in Hu sheep GCs by targeting *INHBA*.

Growth factors and mitogens impact the proliferation state by triggering various cytoplasmic signaling cascades that activate cyclin-dependent kinases (CDKs) to propel the cell cycle forward. P21 and other cyclin-dependent kinase inhibitors (CKIs) are involved in controlling CDK activity [22]. The EDU and CCK-8 assays negatively correlated GC proliferation and miR-134-3p levels. However, the amount of *INHBA* appears to impact how miR-134-3p affects GC growth. miR-134-3p overexpression promoted cell accumulation in the S phase while decreasing G0/G1, which is consistent with the protein levels of PCNA. However, GC proliferation and miR-134-3p levels were positively linked, as evidenced by alterations in the levels of many proliferation-associated genes. These discrepancies may be caused by miR-134-3p’s ability to promote apoptosis, which raises the relative percentages of proliferation in treated GCs, compared to the controls. Recently, a positive association between GC proliferation and *INHBA* expression level has been discovered [10]. By targeting many genes, including *PRSS57*, miR-134-3p can stimulate and inhibit ovarian cancer cell growth [23,24,25]. Due to their numerous targets, miR-134-3p may either stimulate or prevent the proliferation of GCs by influencing several associated genes. In other words, the availability of their target genes can influence how miR-134-3p affects cell growth.

While extensive GC apoptosis leads to follicle demise, it seems to be a standard component of follicle maturation. It indicates the mitogenic proliferation of the follicle, which fluctuates according to developmental stages [26,27]. The results indicated that miR-134-3p enhanced GC apoptosis via *INHBA*. The current study determined that miR-134-3p promoters or inhibitors had a similar efficiency trend on the gene levels of both *BAX* and *BCL2* genes, indicating that, similar to our findings for GCs proliferation, the influence of miR-134-3p on the expression of apoptosis-related genes can be influenced by the availability of their target genes. Furthermore, *BAX/BCL2* was upregulated by the miR-134-3p promoter and inhibitors, whereas *BAX/BCL2* was downregulated by *INHBA* overexpression and knockdown, respectively.

Interestingly, miR-134-3p overexpression and high *CASP8* expression may indicate a mitochondria-independent apoptotic pathway. Previous research has found that miR-134-3p has the opposite effect on cell apoptosis as it does on proliferation, as miR-134-3p induced cell apoptosis in ovarian tumor cells [24], while it inhibited cell apoptosis in other cell models [25,28]. Our former study noted a negative correlation between the *INHBA* expression level and GC apoptosis [10]. Our findings suggest that miR-134-3p can provide appropriate hemostats between GC proliferation and apoptosis during early follicular development by targeting several genes.

*INHBA* encodes a subunit of activin and inhibin. The *PI3K-AKT* axis, which causes mTOR activation, can be activated directly or indirectly by TGF-β signaling [29,30]. In gliomas, miR-134 overexpression prevented the activation of the *PI3K/AKT* axis [31]; this was also the case in colorectal tumor cells [32]. The recruitment of primordial follicles, GC proliferation, corpus luteum formation, and oocyte maturation are all aspects of ovarian follicle development that can be impacted by the stimulation of the *PI3K/AKT* axis [33,34,35,36,37,38]. Low fecundity in Hu sheep is indicated by low expressions of *TGFβ1* and *INHBA*, which may be related to a downregulation of the TGF-signaling pathway [11]. According to our research, miR-134-3p suppressed the levels of many associated genes while increasing those of *TGFβR2*, hence downregulating the PI3K/AKT/mTOR axis. However, the influence of miR-134-3p on *TGFR2* expression was dependent on *INHBA* levels, as miR-134-3p inhibition with low *INHBA* levels promoted both *TGFR2* expression and PI3K/AKT/mTOR signaling, which could indicate a TGF-β pathway independent of miR-134-3p. In summary, miR-134-3p may modulate cytokines in GCs via the PI3K/AKT/mTOR axis, though further investigation is necessary to examine miR-134-3p targets in Hu sheep GCs.

## 5. Conclusions

In summary, the present results highlight the significant function of miR-134-3p in regulating the proliferation and mortality of sheep GCs by focusing on *INHBA* mRNA. The effects are probably mediated via the PI3K/AKT/mTOR axis. However, the evidence indicates that miR-134-3p is a significant non-coding RNA in the follicular development of sheep GCs. Further investigations are required to elucidate its specific mechanisms in follicular development, considering its multiple targets.

## Figures and Tables

**Table 1:** The sequences of INHBA-3′-UTR vector.

Name	INHBA 3′-UTR Mut Sequence (5′-3′)
miR-134-3p-INHBA-wt	GGAGGAGTGTGGCTGCTCATAGAGCGCCCAGCCCAGGGGGGACGGGAGCGAGATGGTCCA
miR-134-3p-INHBA-mut	GGAGGAGTGTGGCTGCTCATAGAGCGCCAGTTAACTGGGGGACGGGAGCGAGATGGTCCA
miR-10b- INHBA-wt	CAACCCCATCCTACAAGTTACTTACTCCCTACAGGGAAAACAAAACAGGAAACTCATGTT
miR-10b- INHBA-mut	CAACCCCATCCTACAAGTTACTTACTCTACGACTTGAAAACAAAACAGGAAACTCATGTT

**Table 2:** miRNA and INHBA interference sequences.

Name	Sequence (5′-3′)
miR-134-3p mimics	Sense: UCUGGGCUGCCUCGUCACCAACC
	Antisense: UUGGUGACGAGGCAGCCCAGAUU
miR-10b mimics	Sense: CAAGUUACUUACUCCCUACAGGG
	Antisense: UGUAGGGAGUAAGUAACUUGUU
NC mimics	Sense: UUCUCCGAACGUGUCACGUTT
	Antisense: ACGUGACACGUUCGGAGAATT
miR-134-3p inhibitor	GGUUGGUGACGAGGCAGCCCAGA
miR-10b inhibitor	CCCUGUAGGGAGUAAGUAACUUG
NC inhibitor	CAGUACUUUUGUGUAGUACAA
siRNA-INHBA	Sense: GGCAGAACAUCAUCAAGAATT Antisense: UUCUUGAUGAUGUUCUGCCTT
siRNA-NC	Sense: UUCUCCGAACGUGUCACGUTT Antisense: ACGUGACACGUUCGGAGAATT

**Table 3:** Sequences of miRNA primers.

Gene	Primer Sequence (5′-3′)
miR-134-3p	F: TCTGGGCTGCCTCGTCA
	R: AGTGCAGGGTCCGAGGTATT
miR-10b	F: GCGACCCTGTAGAACCGAA
	R: AGTGCAGGGTCCGAGGTATT
miR-16b	F: GCGCTAGCAGCACGTAAA
	R: TGGTGTCGTGGAGTCGGCAAT

**Table 4:** Primer sequences used in the present study.

Items	Primer Sequence (5′-3′)	Fragment Size (bp)	R^2^	E
INHBA	F: GGACATACGGATTGCCTGTGAGC	110	0.998	1.96
	R: CTCCATCCCTCTTCTTCCCTTCCC			
GAPDH	F: GTCAAGGCAGAGAACGGGAA	232	0.998	1.99
	R: GGTTCACGCCCATCACAAAC			
SMAD2	F: CTTGAGAAAGCCATCACCAC	90	0.998	1.96
	R: TCGATGGGACACCTGAAG			
BAX	F: CGAGTGGCGGCTGAAAT	286	0.996	1.96
	R: GGTCTGCCATGTGGGTGTC			
BCL2	F: CGCATCGTGGCCTTCTTT	113	0.993	1.92
	R: CGGTTCAGGTACTCGGTCATC			
CASP3	F: TCAGGGAAACCTTCACGAGC	274	0.998	1.96
	R: CCTCGGCAGGCCTGAATAAT			
CAS98	F: TGAAGGTTCCAGGATTCGCC	136	0.996	1.96
	R: GGCTTAGGAACTTGAGGGCA			
CASP9	F: CAGACGGATGTCCTGTGTCC	178	0.995	1.93
	R: GGGTTGCTATTGGGGGTCTC			
P53	F: TTCCCCTTCCCTCAACAAGC	234	0.999	1.94
	R: GCGCGTAAATTCCCTTCCAC			
CDK1	F: ATGGCTTGGATCTGCTCTCG	154	0.999	1.94
	R: TGCTCTTGACACAACACAGGA			
CDK4	F: GCTGCTGCTGGAGATGCTGAC	100	0.996	1.95
	R: CTCTGCGTCACCTTCTGCCTTG			
CCNB1	F: GCTTGGAGACATCGGTAACA	129	0.998	1.98
	R: GGAGCCTTTTCCAGAGGTTTTG			
CCND1	F: ACATGGAGCTGGTCCTGGTGA	188	0.996	1.96
	R: GGAGGGTGGGTTGGAAATGAA			
CCND2	F: AGCACGCTCAGACCTTCATC	193	0.998	1.96
	R: AGGCAATCCACATCCGTGTT			
P1K3CA	F: GAGGAGCCCCGAGCATTTCT	134	0.996	1.95
	R: AAGTGGATGCCCCACAGTTC			
AKT3	F: CAGCAGAGAATCCAAACCCCA	133	0.997	1.96
	R: TCCCTTTACCAGCACCCCTCT			
mTOR	F: CTGGAGGCTGATGGACACAAAT	190	0.996	1.99
	R: CTCTGGTTTCACCAAACCGTATC			
TGFβR2	F: GGTGGGAACGGCGAGATAC	158	0.996	1.98
	R: CGTAGTCCTTCACTTCTCCCACG			

Note: E—efficiency.

**Table 5:** Antibody Specifications.

Antibodies	Cat No.	Source	Dilution of WB
INHBA	T58360	Abmart, Shanghai, China	1:1000
BAX	50599-2-Ig	ProteinTech, Chicago, IL, USA	1:2000
BCL2	12789-1-AP	ProteinTech, Chicago, IL, USA	1:1000
GAPDH	60004-1-Ig	ProteinTech, Chicago, IL, USA	1:8000
PCNA	Ab15497	Abcam, Cambridge, UK	1:500
TGFβR2	27212-1-AP	ProteinTech, Chicago, IL, USA	1:1000
SMAD2	12570-1-AP	ProteinTech, Chicago, IL, USA	1:1000
mTOR	2983	CST, Boston, MA, USA	1:1000
P-mTOR	5536	CST, Boston, MA, USA	1:1000
AKT	60203-2-Ig	ProteinTech, Chicago, IL, USA	1:1000
P-AKT	AF0016	Affinity, Boston, MA, USA	1:1000
Goat Anti-Mouse	SA00001-1	ProteinTech, Chicago, IL, USA	1:5000
Goat Anti-Rabbit	SA00001-2	ProteinTech, Chicago, IL, USA	1:5000

## Data Availability

The data presented in this study are available upon request from the corresponding author.

## Supplementary Materials

The following supporting information can be downloaded at: https://www.mdpi.com/article/10.3390/biology14010024/s1. Figure S1: Original Western bolt (WB) images; Figure S2: Original Western bolt (WB) images; Figure S3: Original Western bolt (WB) images; Figure S4: Original Western bolt (WB) images; Figure S5: Original Western bolt (WB) images.
