# Supplementary material for: miR-134-3p Regulates Cell Proliferation and Apoptosis by Targeting INHBA via Inhibiting the TGF-β/PI3K/AKT Pathway in Sheep Granulosa Cells
Source: Biology (Basel). 2024 Dec 30;14(1):24. doi: 10.3390/biology14010024 (PMC11759767; doi:10.3390/biology14010024)
Supplement: Supplementary file 1 [file biology-14-00024-s001.zip › biology-3336122-supplementary.pdf]

Figure S1

A

INHBA GAPDH

INHBA GAPDH

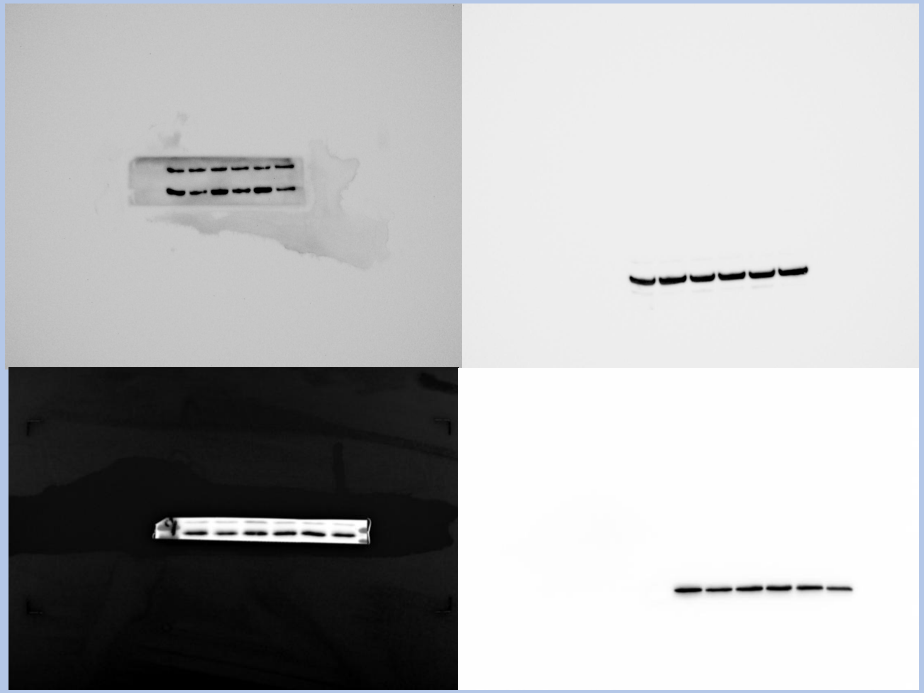

B

INHBA GAPDH

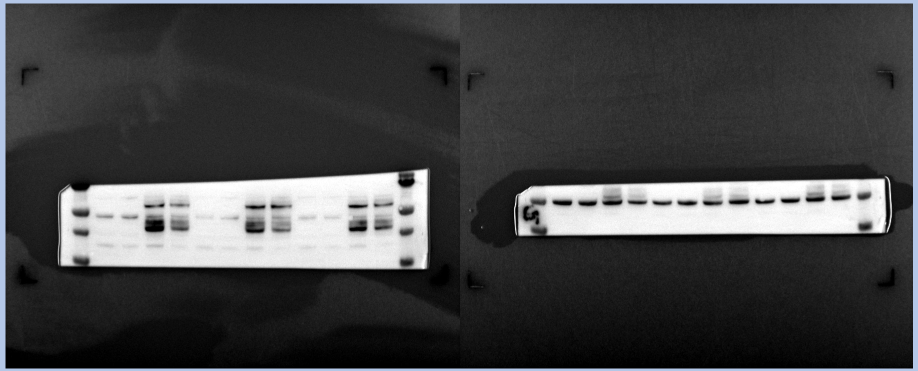

C

INHBA GAPDH

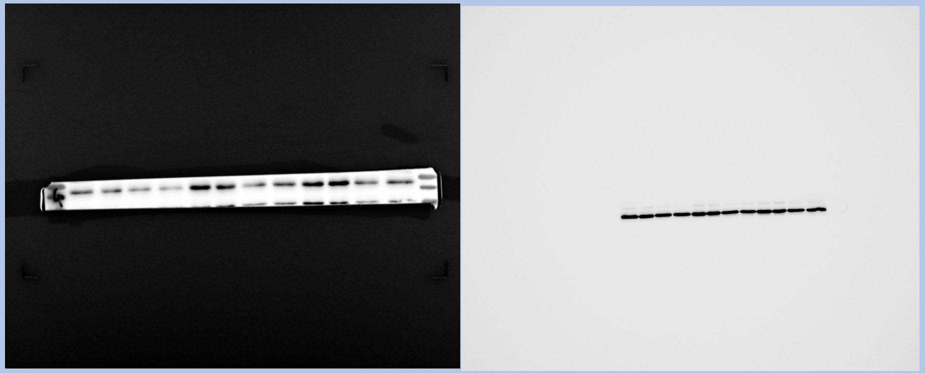

Figure S2

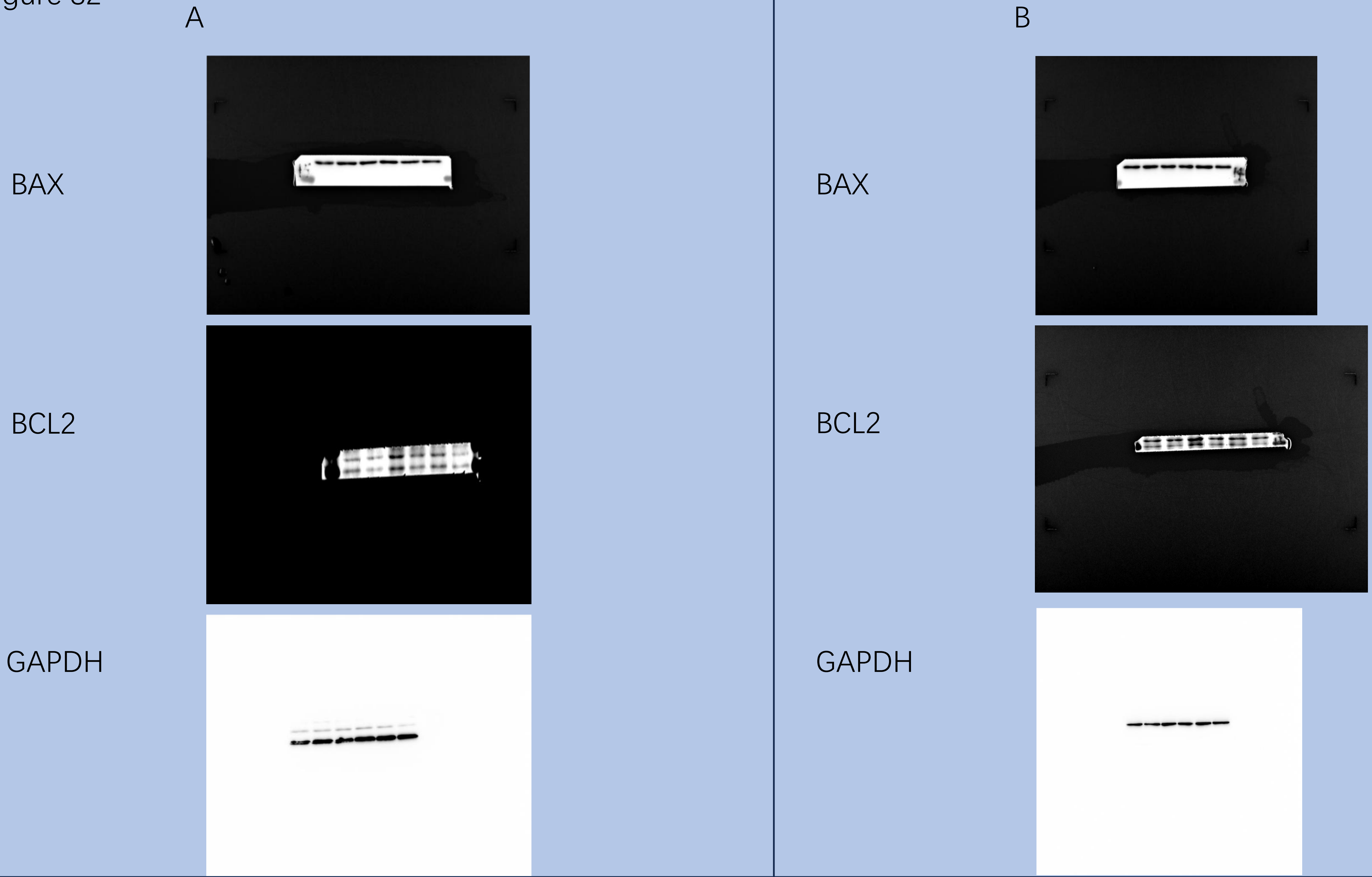

Figure S3

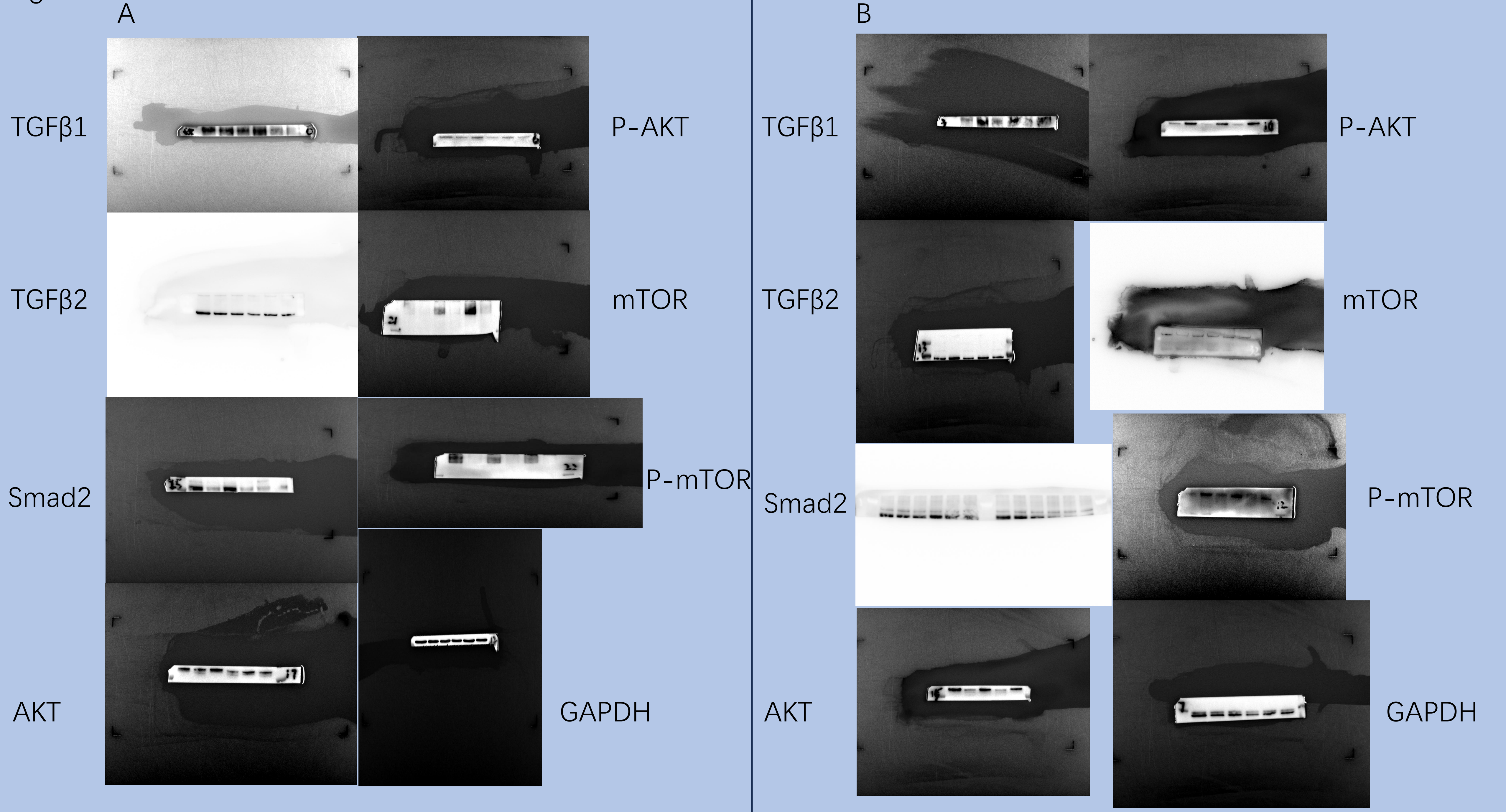
A
TGFβ1
P-AKT
TGFβ2
mTOR
Smad2
P-mTOR
AKT
GAPDH
B
TGFβ1
P-AKT
TGFβ2
mTOR
Smad2
P-mTOR
AKT
GAPDH

Figure S4

A

BAX

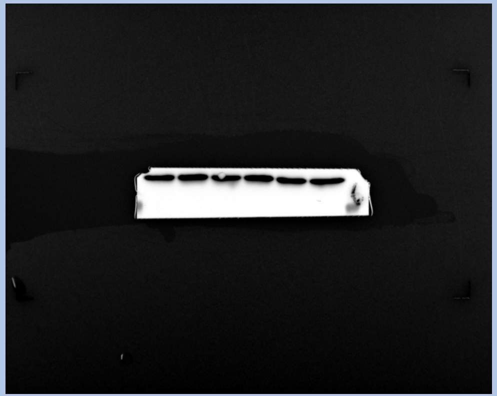

BCL2

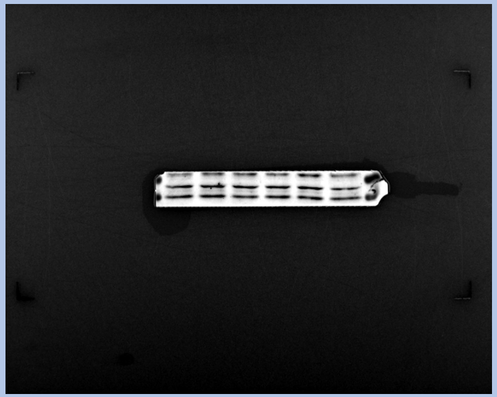

GAPDH

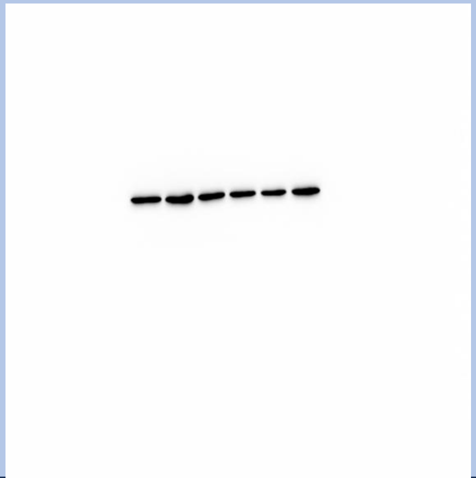

B

BAX

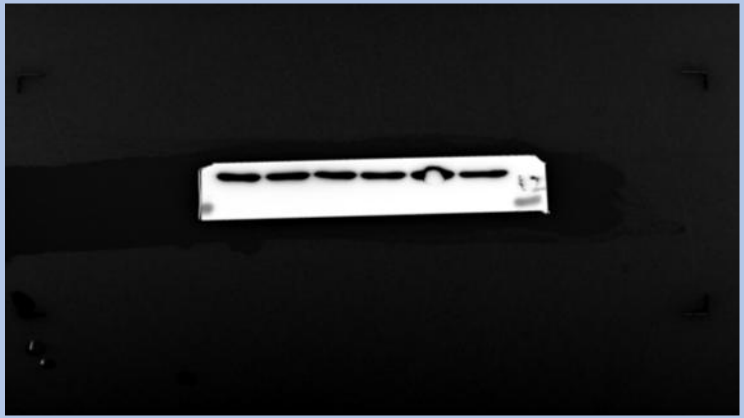

BCL2

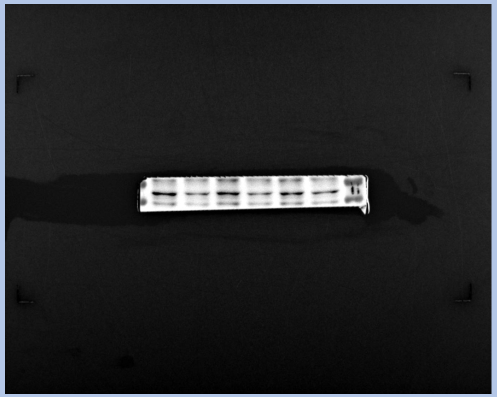

GAPDH

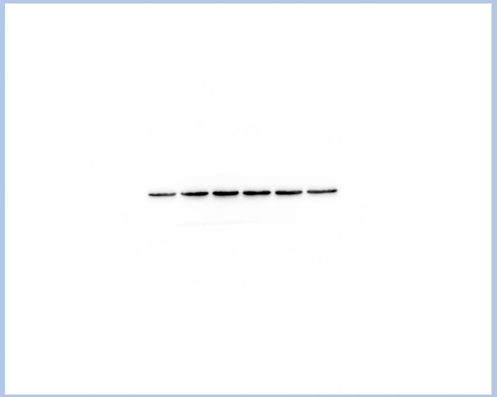

Figure S5

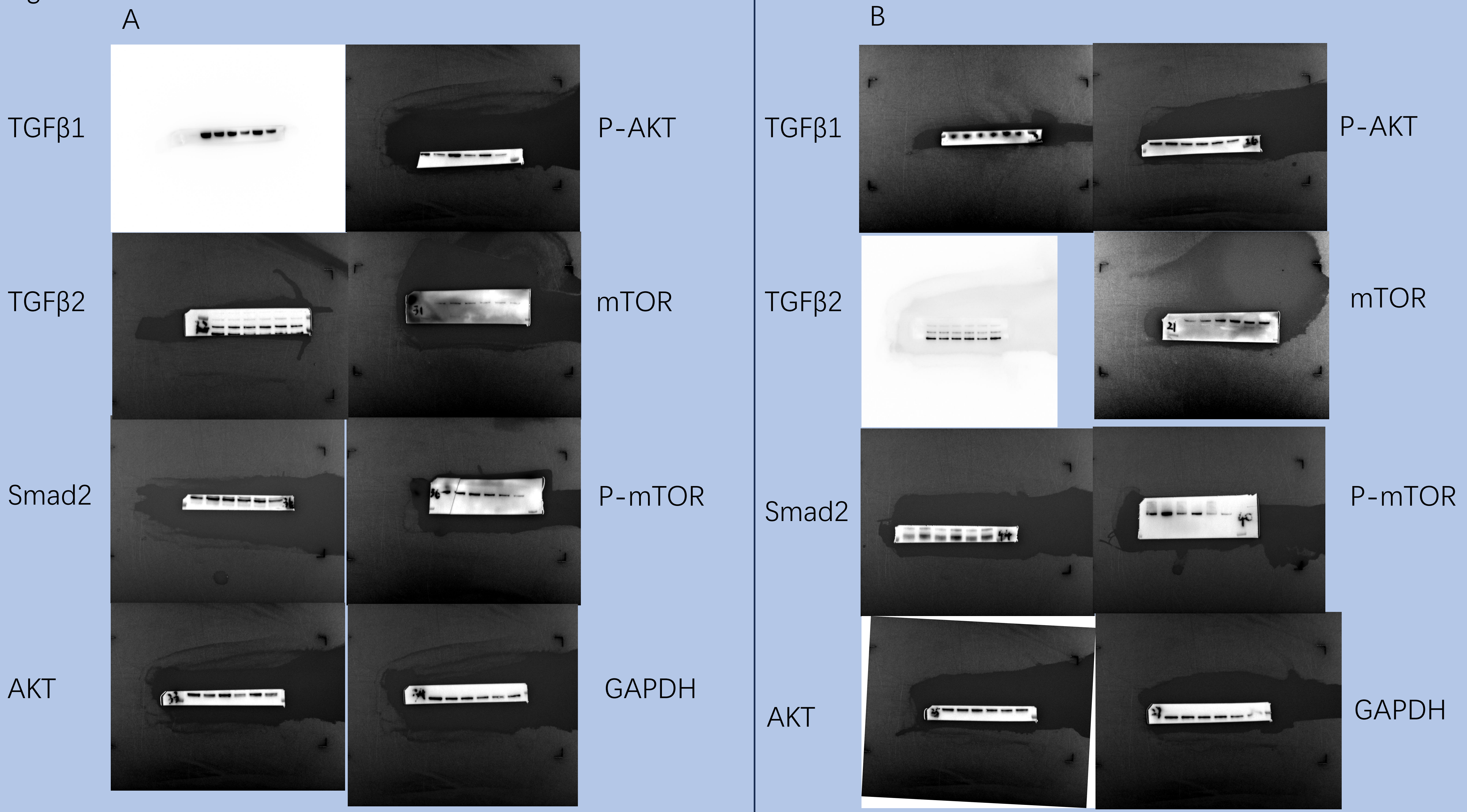
A
TGFβ1
P-AKT
TGFβ2
mTOR
Smad2
P-mTOR
AKT
GAPDH
B
TGFβ1
P-AKT
TGFβ2
mTOR
Smad2
P-mTOR
AKT
GAPDH
